# The basis of S–R learning: associations between individual stimulus features and responses

**DOI:** 10.1007/s00426-023-01873-1

**Published:** 2023-09-18

**Authors:** Willem B. Verwey

**Affiliations:** Department of Learning, Data-Analytics and Technology, Faculty of Behavioural, Management and Social Sciences, University of Twente, PO Box 217, 7500 AE Enschede, The Netherlands

## Abstract

Three experiments tested the hypothesis that response selection skill involves associations between individual stimulus features and responses. The Orientation group in Experiments 1 and 2 first practiced responding to the orientation of a line stimulus while ignoring its color, and the Color group practiced responding to the color of the line while disregarding its orientation. When in the ensuing test conditions the Orientation group responded to the color of the line, RTs and errors increased when the then irrelevant line orientation was inconsistent with practice. This confirmed that during practice, Orientation participants had developed orientation feature–response associations they could not fully inhibit. Yet, evidence for color feature–response associations was not observed in the Color group. This was attributed to orientation identification being faster than color identification, even after having practiced responding to colors. Experiment 3 involved practicing to three line stimuli with unique orientation and color combinations. It showed evidence for the independent development of orientation feature–response associations and color feature–response associations. Together, these results indicate that the typical RT reduction with practice in response selection tasks is caused in part by the capacity of participants to learn selecting responses on the basis of the stimulus feature that becomes available first.

## Introduction

Skilled task performance typically involves the rapid selection of responses (R) to stimuli (S). Traditionally, this high selection speed has been attributed to the development of long-term *associations* between stimulus and response representations in memory (Logan, 1988; Teichner & Krebs, 1974; Welford, 1968) which would gradually replace the application of some abstract stimulus–response (S–R) mapping rule or a general algorithm stored in short-term memory (Duncan, 1978; Logan, 1988). Automaticity of these associations is demonstrated, for example, by response slowing when S–R mappings are reversed (Kramer et al., 1990; Pashler & Baylis, 1991; Shiffrin & Dumais, 1981; Verwey, 1999). Later studies casted doubt on this simple model and attributed selection skill to the development of a broad and versatile *task representation*, like an event file (Hommel, 2019; Hommel et al., 2001) or a task file (Hazeltine & Schumacher, 2016; Schumacher & Hazeltine, 2016). Depending on the type of task, these representations are assumed to include abstract stimulus and response representations, concrete visual and motor features on which those higher-level representations are grounded, as well as representations of the context, the expected feedback of the responses, and the goals and drives. Hence, task representations are fuzzy and highly dependent on the advance setting of task and strategic information. But, once properly prepared, stimulus display allows the virtually automatic execution of the required response (Hommel, 2000).

According to the Theory of Event Coding (TEC, Hommel et al., 2001; also see Hommel, 2019), perceptual and action events are represented in a distributed fashion in event files that include features of stimuli and actions in a common format. This allows action representations to be selected by activating the anticipated sensory consequences of those actions. As the same actions may be used in different tasks, later research found evidence for the notion that the intention to perform a task prompts weighting of task-specific semantic and also perceptual features like color and orientation feature dimensions (Memelink & Hommel, 2013). This suggests that responding to stimuli may allow participants to attend to a specific stimulus feature instead of first identifying the stimulus as a whole.

The event file construct of TEC is largely based on studies involving limited learning (Hommel, 2019) and event files rely heavily on preparatory activity temporarily binding features (Hommel & Colzato, 2009; Memelink & Hommel, 2013). It is obvious, however, that the cognitive system also involves representations that are based on more permanently associated features. Even in TEC actions can be selected only because their sensory action effects are permanently associated with response representations. These associations are believed to follow the principles of Hebbian learning (Hebb, 1949; Lowel & Singer, 1992), and therefore result from the repeated co-activation and binding of representations in short-term memory. It has indeed been argued that temporary binding and long-term associations are rooted in different neurophysiological mechanisms (Herwig & Waszak, 2012; Hommel, 2019). While bindings are economical in terms of long-term memory load and wasteful in terms of processing time, associations are wasteful in terms of long-term memory load and economical in terms of processing time (Hommel & Colzato, 2009).

Inspired by neurophysiological research that distinguished cortical hierarchies of motor functions at the anterior side, and of stimulus representations at the posterior side of the brain (Fuster, 2004; Koechlin & Summerfield, 2007; Koechlin et al., 2003), Schumacher and Hazeltine proposed the task file framework (Hazeltine & Schumacher, 2016; Schumacher & Hazeltine, 2016). Task files span abstract goals, and hierarchical stimulus and response representations. Associative S–R learning would connect elements at task-dependent levels of these stimulus and response hierarchies. So, perceiving the features that make up a stimulus (like its color, orientation, size, shape, location, motion, and depth) elicits a cascade of processes that activates increasingly more encompassing stimulus representations (Treisman & Gelade, 1980; for a review, see, e.g., Humphreys, 2016). Similarly, response representations have been proposed to include motor features like relative timing, relative force and the sequencing of sub-movements that together have been referred to as a general motor program (Schmidt, 1975; Shea & Wulf, 2005). This motor program is then scaled by specifying features, or ‘movement parameters’, like absolute movement time and absolute force. In this task file framework, response selection is the logical consequence of satisfying the constraints provided by the displayed stimulus, the prepared task and goal, the environment, and the organism’s motivational state. This framework accounts for indications that selecting responses may be based on stimulus representations at various hierarchical levels, like categories of stimuli (Pashler & Baylis, 1991), *compound* stimuli, but also individual stimulus features (Neumann, 1990; also see, e.g., Ansorge et al., 1998). The latter possibility was later worked out in the Dimension-Action (DA) Model by the notion that attention-dependent modules process specific visual features and that each of these modules possesses its own response selection mechanism (Magen, 2019; Magen & Cohen, 2002; for neural indications, see Jeannerod, 1997; Pisella et al., 2006).

### The present study

The reported research was designed to test the *stimulus feature–response association* hypothesis. This hypothesis asserts that, in contrast to the classic S–R association model, response selection skill is based on associations between the lowest possible levels of the perceptual and motor hierarchies (Fuster, 2004). At the perceptual side, the representations at these lower levels are probably also available the fastest. At the motor side, they are likely to distinguish the required response from the other responses in the prepared response set (Cisek, 2007; Neumann, 1990). A diminishing need with practice in choice-RT tasks to identify a stimulus as a whole would contribute to the typical RT reduction with practice to select responses. Participants indeed appear able to focus on a specific stimulus feature and ignore other stimulus features (Magen, 2019; Magen & Cohen, 2002; Müller et al., 2003; Su et al., 2014; Wolfe, 2021). Given that these associations are task specific, this hypothesis predicts for instance that when, after practicing responding to the orientation of a line stimulus, participants respond to its color, these responses are slowed when the then irrelevant line orientation is associated with another response. The RT difference between responses to stimuli that include an irrelevant feature that is inconsistent, as opposed to consistent, with responses practiced before is called here the *consistency effect*.

Some visual features are identified more rapidly than others (Eimer et al., 1995; Miller, 1988). The location feature of a stimulus would be identified more rapidly than its color (Pisella et al., 1998), color would be identified faster than a letter (Miller, 1982; Smid et al., 1992), color and line orientation would be identified more rapidly than shape (Su et al., 2014), and stimulus location would be identified before a letter or digit (Osman et al., 1992). Together, these studies suggest that location is available more rapidly than color and orientation, and these would be available before more complicated stimulus shapes like letters, squares and circles.

In the present study, the stimulus feature–response association hypothesis was tested in three experiments in which participants practiced responding to either the color or the orientation of three line stimuli. These features were used because they seemed to take about as long to identify. Experiments 1 and 2 explored the development of color–response and orientation–response associations during practice. Experiment 3 investigated whether color–response and orientation–response associations remain independent when during practice the color and orientation of the stimuli have been fully confounded. In short, the experiments reported below supported the stimulus feature–response association hypothesis for orientation–response associations. The evidence for color–response associations was more limited, probably because identifying the color of a line stimulus takes more time than identifying its orientation, but both types of association seem to remain independent.

## Experiment 1

In the first experiment, participants practiced a 3-choice RT task in which line stimuli were displayed with 1 of 3 alternative orientations (right tilted line or slash: ‘/’, vertical line: ‘|’, left-tilted line or backslash: ‘\’) in 1 of 3 alternative colors (red, green, blue). The *Color group* practiced responding to the line color and the *Orientation group* to the line orientation. It was hypothesized that the Color group would develop an association between the color feature and the response, and the Orientation group would develop an association between the orientation feature and the response. Development of these associations was assessed in the test phase by requiring participants of both groups to respond to the same 9 stimuli (3 line orientations displayed in 3 colors) but now with the instruction of the other participant group. Consequently, responding to the line orientation implied that the Color group performed trials in which the—now irrelevant—color was consistent, and trials in which the color was inconsistent, with the responses previously practiced with. In a similar vein, when the Orientation group responded to line colors in the test condition they performed trials with consistent and inconsistent line orientations. The stimulus feature–response association hypothesis predicts that in the test condition, RTs are longer with an inconsistent than with a consistent stimulus feature because the irrelevant stimulus feature automatically primes another than the required response, thus causing a response conflict.

### Method

#### Participants

Forty-eight students from the University of Twente participated in exchange for course credits or financial reward. This number was based on findings in a pilot study. A post hoc power analysis using GPower 3.1.9.2 (Faul et al., 2007) based on the result with the most important effect of the test phase (i.e., utilizing sample size, number of groups, number of trials, and effect size of the Practice Group × Target Feature × Consistency interaction on RTs reported in the test phase) showed that with 16 participants per group (given 0.05 alpha, and two-tailed testing) a power would have been reached of 0.95 for detecting this effect. Participants were included in the experiment only when they were not heavy smokers and had not consumed alcohol in the twenty-four hours prior to the experiment to prevent performance from being influenced by withdrawal symptoms and adverse effects of alcohol. The participants were randomly distributed across the Color group (13 female and 11 male, 18–44 years old, mean age 23.0 years) and the Orientation group (14 female, 10 male; 17–25 years old, mean age 22.2 years). This study was approved by the ethics committee of the Faculty of Behavioural, Management, and Social Sciences at the University of Twente.

#### Apparatus

The experiment ran on a Dell 2.8 GHz Pentium 4 PC with Windows 7. E-Prime 2.0 was used for running the experiment. Unnecessary Windows services were shut down to improve the measurement of response times (RTs). The stimuli were presented on a 22-inch LG Flatron E2210PM-BN LCD screen applying the 1280 × 1024 pixel resolution, using a Logitech Deluxe 250 USB keyboard. Stimuli consisted of three Courier New characters in EPrime with size 24 (backslash ‘\’: -26.5° left-tilted, height: 8 mm; vertical line ‘|’: 0°, height: 7 mm; slash ‘/’: 26.5° right tilted, height: 8 mm). Their colors were the predefined EPrime colors which were either red (RGB: 255,0,0), green (0,128,0) or blue (0,0,255). Viewing distance was approximately 50–60 cm and not controlled. Progress of the experiment was monitored via a GoPro observation camera.

#### Task

Participants positioned the index, middle, and ring fingers of their dominant hand on the keys J, K, and L on the keyboard. Each trial started with the display of the colored line stimulus. This stimulus was erased upon response onset. If the response was correct it was followed by clearing the display for 800 ms. In the case of an error, an error message was displayed for 800 ms. If no response had been given after 10 s, this was indicated. Then, the next stimulus was displayed, and so on.

Participants in the Color group practiced by responding as fast as possible to the stimulus color while ignoring its orientation (see Table 1). They pressed the J key to the three red (‘|’, ‘/’, and ‘\’) stimuli, the K key to the same three line stimuli in green, and the L key to these stimuli in blue. Participants in the Orientation group practiced by reacting to the orientation of the line, ignoring its color. They pressed J to the slash (‘/’) stimuli, K to the three colored backslash (‘\’) stimuli, and L to the three vertical line stimuli (‘|’). The 9 different stimuli occurred equally often in each practice block. Practice included 9 blocks with 198 trials each, 22 trials with each stimulus yielding 198 practice trials with each of the 9 stimuli. The order of the 9 S–R combinations was randomized in each practice block.

**Table 1:**
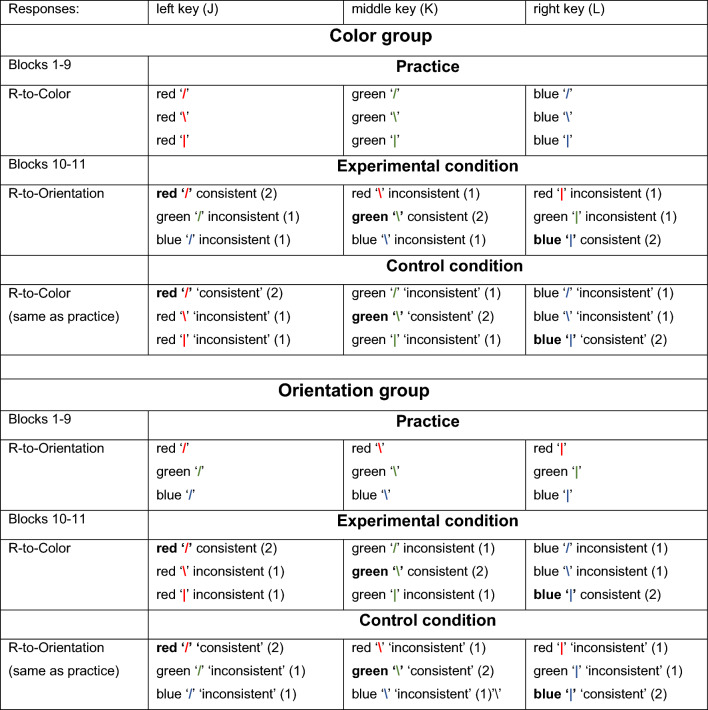
The stimulus–response assignments of the Color and the Orientation practice groups in Experiment 1

(1) In the Experimental conditions of the Color and the Orientation groups the consistent trials consisted of the same S–R combinations as during practice and the inconsistent trials consisted of stimuli that during practice had required another response. (2) In the Control conditions of Blocks 10 and 11 the same stimuli were considered consistent as in the Experimental condition while the other 2 stimuli were considered ‘inconsistent’. However, as all stimuli in the Control condition had been practiced a consistency effect was not expected in the Control condition. (3) The bracketed numbers indicate the relative trial frequencies in the test blocks. Doubling the likelihood of consistent trials in the test conditions ensured an equal number of inconsistent and consistent trials in each trial block

The ensuing test phase included two identical test blocks, Blocks 10 and 11. Each of these included two 72-trial subblocks, one containing the Control condition and the other the Experimental condition (Table 1). The order of these two subblocks was counterbalanced across the participants of each group within Blocks 10 and 11. In the Experimental condition, the same stimuli were displayed as during practice but participants responded to the stimulus feature they had been ignoring during practice. So, the Color group reacted to the orientation of the line and the Orientation group reacted to the line color. Accordingly, in the Experimental condition the 9 S–R combinations included consistent and inconsistent responses. For the Color group responding with the J key to the red ‘/’, with K to the green ‘\’, and with L to the blue ‘|’ was consistent with practice. The other responses were inconsistent with practice because the color had previously evoked another response. Similarly, the Orientation group included consistent and inconsistent responses in the Experimental condition (Table 1). The task in the Control condition was identical to the one practiced and, hence, included only consistent trials. To make sure that any consistency effect could not be attributed to specific stimuli, the consistent stimuli in the Experimental condition of this experiment were denoted consistent in the Control condition too while the other stimuli were denoted inconsistent. Yet, in both practice groups, the control condition was not expected to yield a consistency effect because the so-called inconsistent trials had been practiced too.

As 3 of the 9 alternative trials in this block were consistent and 6 were inconsistent, a 50/50 likelihood on consistent trials was achieved by presenting the consistent trials twice as often as the inconsistent trials. The two test blocks were separated by a 60-s pause. Practice and test blocks ended by displaying for 6 s the mean response time and the percentage of errors the participants had made. This was always accompanied by the instruction to limit errors to (an arbitrary) 8%.

#### Procedure

The experiment took place in two cubicles equipped with a computer and a video camera allowing participant monitoring. The participants sat down in front of the computer and filled out an informed consent form. The experimenter explained the task to the participant, that detailed instructions would be given on the display, and that after the break following each block the experimenter would enter and start the next block. The Ishihara color test was administered to assure that participants did not have a form of color blindness (Ishihara, 1918, 1996). Each trial block was followed by performance feedback in terms of mean RT and error percentage. It was explained that with more than 20% error trials the computer would repeat the block (eventually this happened only once). Then the first practice block was started by the experimenter. Before the first test block, the participants were told that in one subblock the task would change and that they were to make sure they followed the instructions. Blocks were separated by a 120-s computer-controlled break after which the experimenter entered the room and started the next block. For individual participants, the overall experiment took 70–80 min.

### Results

#### Practice phase

**Fig. 1:**
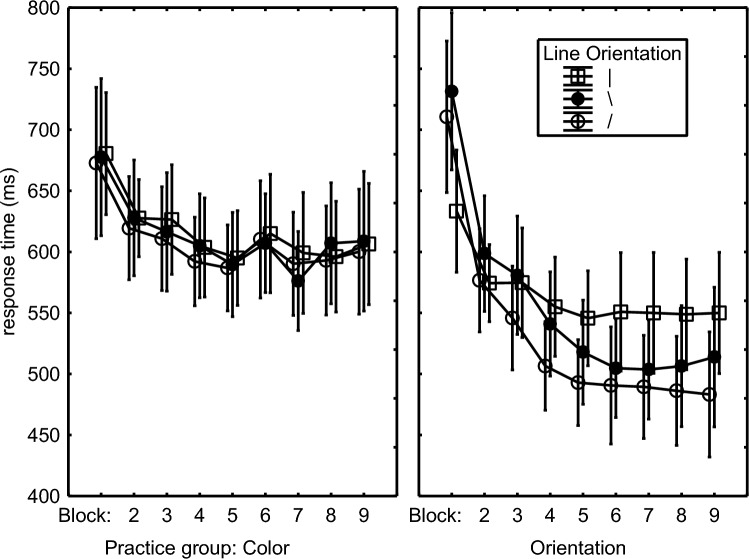
Response times in the Color and Orientation groups for the three line orientations. The error bars in this figure and in the ones below indicate the standard error of the means.

The RTs from errorless trials in Blocks 1–9 were analyzed with a mixed 2 (Practice Group: Color group vs. Orientation group) × 3 (Color: blue, green, red) × 3 (Orientation: slash, backslash, vertical line) × 9 (Block: 1–9) ANOVA with Practice Group as between-subject variable. It showed a significant Block main effect, *F*(8,368) = 28.64, *p* < .001, *η*_p_^2^ = .38, and a marginally significant Practice Group effect (613 ms vs. 551 ms), *F*(1,46) = 3.66, *p* = .06, *η*_p_^2^ = .07, that together with a Practice Group × Block interaction, *F*(8,368) = 4.50, *p* < .001, *η*_p_^2^ = .09, indicated an RT decrease with practice that leveled off earlier for the Color than for the Orientation group (Fig. 1, Orientation benefit over Color: Block 1: − 5 ms, Block 2: 42 ms, Block 9: 89 ms) and that eventually yielded faster response in the Orientation than the Color group.

The Practice Group × Block × Orientation interaction, *F*(16,736) = 5.04, *p* < .001, *η*_p_^2^ = .10, together with the Orientation main effect, *F*(2,92) = 4.70, *p* = .01, *η*_p_^2^ = .09, showed that this later levelling off in the Orientation group involved an increasing difference between line orientations during the first about 4 blocks, *F*(2,92) = 18.19, *p* < .001, *η*_p_^2^ = .28. This effect of line orientation was not observed in the Color group, *F*(2,92) = 0.05, *p* = .95. Conversely, no statistically significant RT differences were observed of the three colors of the lines in the Color group (varying between 594 and 601 ms) and neither in the Orientation group (between 515 and 517 ms), *F*s(2,92) < .21, *p* > .80.

Error proportions were arcsine-transformed before being submitted to an ANOVA to stabilize the variance (see p.356 in Winer et al., 1991). This ANOVA involved the same design as the above RT analysis. A Practice Group × Block interaction showed that error proportion slightly increased for the Color group across successive practice blocks (Block 1: 3.8% vs. Block 8: 5.5%, Block 9: 4.5%) while it slightly decreased in the Orientation group (Block 1: 5.0%, Block 8: 3.6%, Block 9: 3.7%), *F*(8,368) = 3.97, *p* < .001, *η*_p_^2^ = .08.

Like with the RTs, this ANOVA further showed for the Orientation group effects of line orientation but this time an effect of color also developed during practice in the Color group. The effect of line orientation in the Orientation group was indicated by a main Orientation effect, *F*(2,92) = 3.73, *p* = .028, *η*_p_^2^ = .08, which according to the Practice Group × Block × Orientation interaction, *F*(16,736) = 2.45, *p* = .001, *η*_p_^2^ = .05, was larger in the Orientation than the Color group and increased with practice. Indeed, planned comparisons showed that line orientation effects differed only in the Orientation group across Blocks 5-9: ‘/’: 2.4%, ‘\’: 3.7%, ‘|’: 4.2%, *F*(2,92) = 12.65, *p* < .001, *η*_p_^2^ = .22, while line orientation did not affect errors in the Color group (across Blocks 5–9: ‘/’: 4.8%, ‘\’: 4.6%, ‘|’: 5.1%, *F*(2,92) = 1.03, *p* = .36). In a similar vein, color affected error proportion in the Color group and not in the Orientation group. That is, across Blocks 5–9, different colors yielded somewhat different error proportions in the Color group (blue: 3.9%, green: 5.3%, red: 5.4%), *F*(2,92) = 4.47, *p* < .01, *η*_p_^2^ = .09, while color had no effect on errors in the Orientation group (across Blocks 5–9: blue: 3.4%, green: 3.5%, red: 3.4%), *F*(2,92) = 0.0, *p* = .99.

In summary, RT reduced across more blocks for the Orientation than for the Color group and was eventually shorter for the Orientation group. In the course of practice responses of the Orientation group to slashes became fastest and involved the lowest error proportion while responses to the vertical lines were relatively slow and involved most errors. Conversely, in the Color group, responses to blue lines involved less errors than to green and red lines, but this color effect was not observed in the RTs.

#### Test phase

RTs of the accurate responses were analyzed with a mixed 2 (Practice Group: Color vs. Orientation) × 2 (Target Feature: Color vs. Orientation) × 2 (Consistency: of the irrelevant feature with practice) × 2 (Block: 10 vs. 11) ANOVA with Practice Group as between-subject variable. To make the Control condition similar to the Experimental condition, it also distinguished consistent and inconsistent stimuli. That is, in the Experimental and the Control conditions, the same stimuli were considered consistent (see Table 1). The remaining stimuli were considered inconsistent, but in the Control condition these so-called inconsistent stimuli had actually been practiced too and no consistency effect was expected there.

**Fig. 2:**
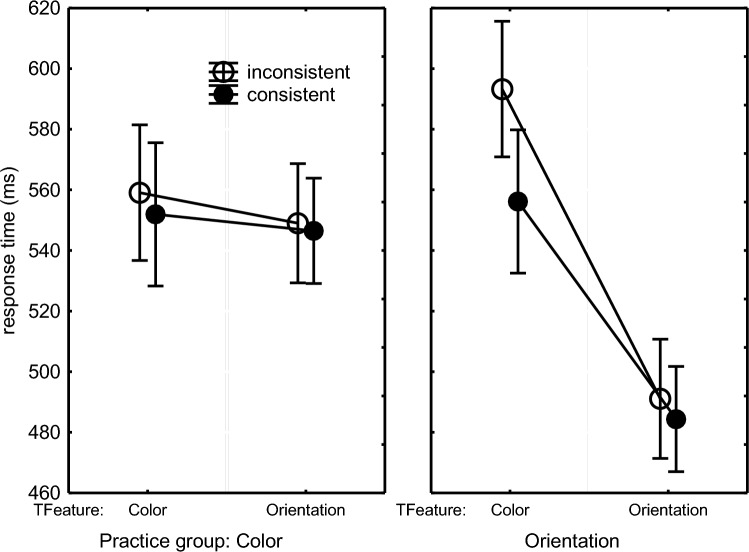
Mean response times in the Color and Orientation practice groups in Experiment 1 as a function of the target feature (‘TFeature’) participants responded to, and whether the irrelevant feature was consistent or inconsistent with practice. In the Color practice group in the left frame, consistency with practice could affect only the responses to the line orientation (right in left frame) as for these Color participants responding to the line color target feature (left in left frame) was always consistent with what they had practiced before. Similarly, in the Orientation practice group in the right frame, consistency with practice affected only the Color condition (left in right frame) as all trials in the Orientation test condition (right in right frame) were consistent with what these participants had practiced before (see Table 1).

The Practice Group × Target Feature × Consistency interaction, *F*(1,46) = 3.83, *p* = .056, *η*_p_^2^ = .01, along with the lower-order interactions Target Feature × Consistency, *F*(1,46) = 7.06, *p* = .01, *η*_p_^2^ = .13, Practice Group × Consistency, *F*(1,46) = 9.52, *p* = .003, *η*_p_^2^ = .17, and Practice Group × Target Feature, *F*(1,46) = 17.48, *p* < .001, *η*_p_^2^ = .28, and a main effect indicating slowed execution of inconsistent responses in general, *F*(1,46) = 23.31, *p* < .001, *η*_p_^2^ = .34, confirmed the prediction that the consistency effect would occur when the Orientation group was responding to color in the Experimental condition and not when responding to the line orientation in the Control condition (Fig. 2). Planned comparisons showed significance of the interaction in the Orientation group (right frame of Fig. 2), *F*(1,46) = 10.65, *p* = .002, *η*_p_^2^ = .19. So, when responding to the line color, responses were slower when the then irrelevant line orientations were inconsistent with practice and had previously triggered another response than when they were consistent and had triggered the same response, *F*(1,46) = 28.36, *p* < .001, *η*_p_^2^ = .38. The Practice Group × Target Feature × Consistency interaction did not reach statistical significance in just the first test block (Block 10), *F*(1,46) = 0.61, *p* = .44, while it did in the second test block (Block 11), *F*(1,46) = 4.70, *p* = .04, *η*_p_^2^ = .09, as if Orientation participants had been better able to overrule the invalid response tendency in Block 10 than in Block 11. In contrast, there was no interaction for the Color group (left frame of Fig. 2) implying that when the Color participants responded to line orientation the line color did not affect responding.

A Practice Group × Block interaction, *F*(1,46) = 12.52, *p* < .001, *η*_p_^2^ = .21, along with a Practice Group main effect, *F*(1,46) = 25.0, *p* < .001, *η*_p_^2^ = .35, and a Block main effect, *F*(1,46) = 13.03, *p* < .001, *η*_p_^2^ = .22, showed a RT reduction across Blocks 10 and 11 in the Color Group (569 ms vs. 534 ms) that was not observed in the Orientation group (531 ms vs. 531 ms).

Finally, a planned comparison of the unfamiliar versus familiar feature in the consistent conditions of each practice group showed that the effect of responding to the unfamiliar feature was significantly greater in the Orientation than in the Color group (Fig. 2), *F*(1,46) = 14.76, *p* < .001, *η*_p_^2^ = .24. Whereas responding to the unfamiliar (color) feature in the Orientation group slowed responding considerably relative to the familiar (orientation) feature (484 ms vs. 556 ms), *F*(1,46) = 25.36, *p* < .001, *η*_p_^2^ = .36, there was no significant slowing when the color group responded to the unfamiliar (orientation) than to the familiar (color) feature (552 ms vs. 546 ms), *F*(1,46) = 0.15, *p* = .70.

**Fig. 3:**
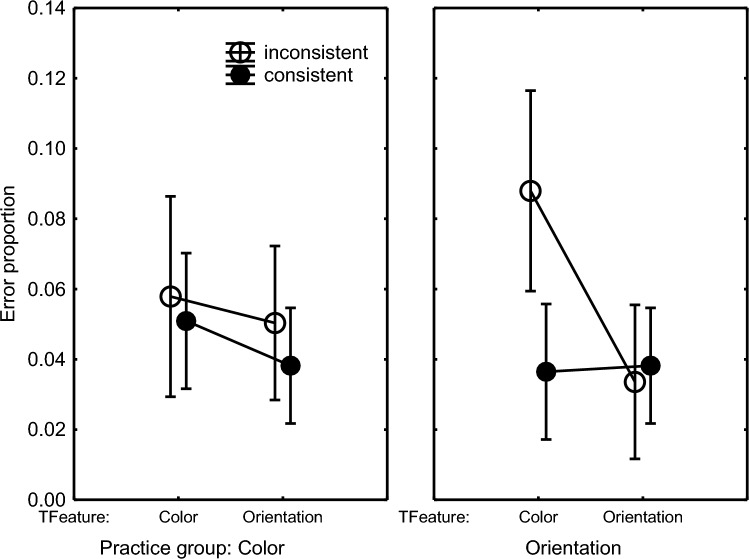
Error proportions In Blocks 10 and 11 in Experiment 1 as a function of the target feature (‘TFeature’) participants responded to, and whether the irrelevant feature was consistent or inconsistent with practice (see remarks in the caption of  Fig. 2)

The errors in the test phase were analyzed by subjecting arcsine transformed error proportions to the above ANOVA design. These effects were entirely in line with the consistency effect occurring only in the Orientation group with the RTs (Fig. 3). That is, the error proportion when the Orientation group was responding to color was higher when the irrelevant line orientations were inconsistent as opposed to consistent. When responding to orientation, there was again no consistency effect. And like with RTs, the consistency effect in error proportion was not statistically significant in the Color group. This group difference was statistically supported by a Practice Group × Target Feature × Consistency interaction, *F*(1,46) = 15.29, *p* < .001, *η*_p_^2^ = .25, which was responsible also for the significant Target Feature × Consistency interaction, *F*(1,46) = 10.20, *p* = .002, *η*_p_^2^ = .18, the Consistency main effect, *F*(1,46) = 10.52, *p* < .001, *η*_p_^2^ = .19, and the Practice Group main effect showing a higher error proportion in the Color than in the Orientation group (5.8% vs. 4.0%), *F*(1,46) = 15.43, *p* < .001, *η*_p_^2^ = .25. Planned comparisons confirmed the interaction for the Orientation group in the right frame of Fig. 3, *F*(1,46) = 25.23, *p* < .001, *η*_p_^2^ = .35, and that the error difference between consistent and inconsistent responses in the Color condition of the Orientation group was significant, *F*(1,46) = 29.24, *p* < .001, *η*_p_^2^ = .39. There was no significant interaction for the Color group (left frame of Fig. 3).

Hence, the test phase showed a consistency effect for the Orientation group in that both RTs and error proportions were higher when these participants were responding to color and the, then irrelevant, line orientation was inconsistent as opposed to consistent with prior practice. This consistency effect appeared to be significant in Block 11 and not in Block 10. Instead, a consistency effect of line color was not found for the Color group in RTs and errors when the participants were responding to the line orientation.

### Discussion

The purpose of Experiment 1 was exploring whether practice induces long-term associations between individual stimulus features and response representations. This was tested separately for the orientation and color features of line stimuli. The development of an association between the line orientation feature and a response representation was supported by the consistency effect found with the Orientation group. This effect involved the response slowing when this participant group was responding to colors in the test condition and the then irrelevant line orientation did not match the response they had previously practiced. This consistency effect was significant in just the second test block and not in the first as if the Orientation participants initially were able to suppress the then irrelevant orientation feature, but then became fatigued and less successful suppressing the irrelevant feature. This initial suppression may have involved the intentional focusing of attention to the color feature while ignoring the then irrelevant line orientation feature (Memelink & Hommel, 2013; Müller et al., 2003; Su et al., 2014).

When the Color group was responding to the line orientation in the test phase the irrelevant color feature did not induce a consistency effect in RT and error proportion. The fact that this effect did not emerge in the Color group suggests that, in contrast to the expectation in the Introduction, color information became available after the response had already been selected on the basis of the orientation feature. A slower identification of the color than of the orientation feature is supported by two findings. First, in the practice phase, responses to color features were generally slower than to line orientations, especially after some practice. Second, for the Orientation group in the test condition responding to the familiar (orientation) feature in the consistent condition was considerably faster (by 72 ms) than to the unfamiliar (color) feature. This can be attributed to the faster identification of the familiar orientation than of the unfamiliar color feature after practice. However, while the Color group most likely benefited also from practice in identifying the stimulus color, eventually the responses of the Color group to the familiar (color) feature were still not faster than to the unfamiliar (orientation) feature (-6 ms). This pattern of results supports that the consistency effect was not found because identifying color is generally slower than identifying line orientation.

The results confirm that during practice participants were attending only to their target feature (cf. Magen, 2019; Magen & Cohen, 2002; Memelink & Hommel, 2013; Müller et al., 2003; Wolfe, 2021). That is, in the course of practice, the Orientation group became slower and made more errors responding to the vertical line than to the two tilted lines (the slashes), while this was not observed with the Color group. Conversely, planned comparisons showed higher error proportions in the later practice blocks for the green and red than the blue lines in the Color group and this was not observed for the Orientation group. This is important also because it shows that participants could ignore irrelevant stimulus features during practice while the consistency effect in the test condition demonstrates that they could not ignore the stimulus feature they had been practicing with before. Hence, only after practice processing of the target feature became mandatory.

## Experiment 2

Experiment 2 was aimed at replicating in a somewhat different task the consistency effect observed in Experiment 1 and examined whether it would again occur in the Orientation and not in the Color group. The task was changed in three ways. First, in Experiment 1, the test phase had involved a 50/50 chance on inconsistent trials by doubling the frequency of the consistent trial (see Table 1). This may, however, have caused faster consistent than inconsistent responses because it may have stimulated participants to prepare for the more frequently occurring consistent response than for the less likely inconsistent responses. To counteract potential effects of response preparation in Experiment 2 the three responses occurred equally often.

The second change concerned the mapping between stimulus and response. In Experiment 1, the same response had been given by all participants to a particular color or line orientation. However, this may have elicited unwanted spatial compatibility effects between especially the line orientation and a particular response (e.g., between the ‘|’ and the L key at the right). Such inadvertent compatibility effects may have caused the performance differences in the practice phase between the individual line orientations in the Orientation group and between the colors in the Color group. Experiment 2, therefore, involved responses consisting of rapidly pressing a single key either 1, 2, or 3 times.

Third, the mapping of the stimuli to these 3 responses was counterbalanced across participants to prevent any potential relationships between stimulus color and orientation, the consistency, and the responses.

Using responses consisting of repeated key presses is interesting also because the effect of a more slowly available irrelevant stimulus feature may emerge after the response key has been pressed for the first time. This might still show a consistency effect in the Color group when responding to line orientation. This possibility is suggested by the many studies showing that even in practiced keying sequences responses can be selected while earlier key presses are being executed (Verwey, 1995, in press; Verwey et al., 2015). Such concurrent response selection could, for example, reduce the size of the consistency effect because key presses may be added to an already executed response consisting of one or two key presses while a selected pressing series may be stopped prematurely.

### Method

Experiment 2 was largely the same as Experiment 1 and involved two groups of 24 participants too. The design again included a Color group (age range 19–27, 15 females, mean 21.1) and a Orientation group (age range 19–27, 17 females, mean 20.8). However, the three responses no longer consisted of pressing three different keys but a single (the ‘G’) key was pressed one, two, or three times. The maximum allowed time between onset of successive key presses was 400 ms. Pressing the key for the wrong number of times, or with an inter key press time of over 400 ms, yielded an error message. These responses were counterbalanced across participants so that overall a specific response was not associated with a particular color or orientation. Here, too, stimuli consisted of the Courier New characters ‘\’, ‘/’, and ‘|’ displayed in red, green or blue.

Experiment 2 again included 9 198-practice trial blocks. The two subblocks of test Blocks 10 and 11 included 54 trials each, and their order was counterbalanced across participants. One subblock contained the control condition which again involved the same task as the practice condition. The other subblock contained the experimental condition in which participants responded to the ‘other’ feature—orientation for the Color group, color for the Orientation group. All nine different stimuli (3 colors × 3 orientations) occurred equally often in each subblock. So, unlike Experiment 1, the experimental subblocks of Blocks 10 and 11 included 36 (67%) inconsistent and 18 (33%) consistent trials. Every participant worked for approximately 120 min.

Stimuli were displayed on an AOC Free Sync 144 Hz monitor which was connected to a Dell Optiplex 7050 computer equipped with a fast PS/2 keyboard and running Windows 10. This time, participants gave their mobile phones for the duration of the experiment to the experimenter to prevent distraction by even the mere presence of their phone (e.g., Thornton et al., 2014).

### Results

### Practice phase

**Fig. 4:**
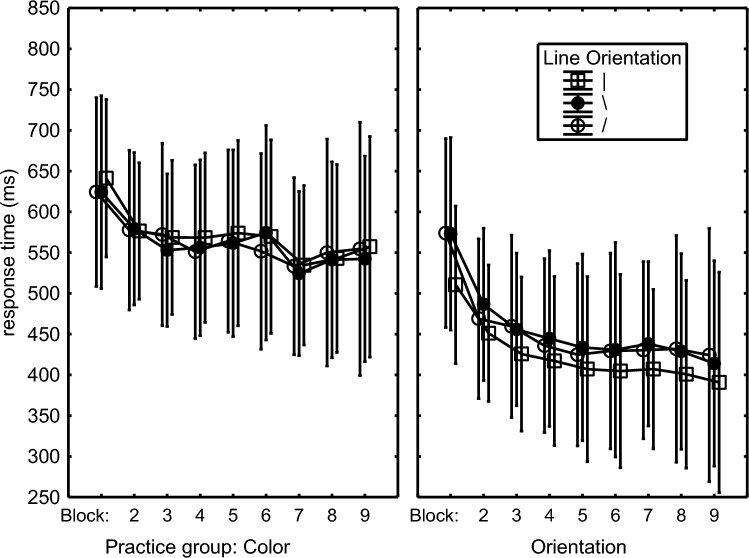
Response times in the Color and Orientation groups for the three line orientations

The RT results were analyzed with two ANOVAs. The first ANOVA analyzed RTs of the first key press of errorless responses in Blocks 1–9 with the same 2 (Practice Group: Color group vs. Orientation group) × 3 (Color: blue, green, red) × 3 (Orientation: slash, backslash, vertical line) × 9 (Blocks: 1–9) ANOVA as in Experiment 1. It showed by way of a Block main effect, *F*(8,368) = 20.63, *p* < .001, *η*_p_^2^ = .31, a Practice group main effect (444 ms vs. 566 ms), *F*(1,46) = 8.14, *p* = .006, *η*_p_^2^ = .15, and a Practice Group × Block interaction, *F*(8,368) = 1.99, *p* < .05, *η*_p_^2^ = .04, that the Orientation group was generally faster than the Color group and that RTs reduced more rapidly in the Orientation than the Color group (Fig. 4).

A Practice Group × Orientation interaction, *F*(2,92) = 6.62, *p* = .002, *η*_p_^2^ = .13, showed that across all practice blocks, responses to the three line orientations were more different for the Orientation group (slash: 453 ms, backslash: 456 ms, vertical: 424 ms) than for the Color group (slash: 570 ms, backslash: 562 ms, vertical: 564 ms). Planned comparisons confirmed that the difference between the three line orientations in the Orientation group was significant, *F*(2,92) = 8.60, *p* < .001, *η*_p_^2^ = .16, while it was not for the Color group, *F*(2,92) = 0.52, *p* = .60. Responses to the vertical line were fastest, instead of slowest like in Experiment 1. This time there was a marginally significant main effect that suggested that RTs differed for the three different colors (green: 510 ms, blue: 506 ms, red: 499 ms), *F*(2,92) = 2.63, *p* = .07, *η*_p_^2^ = .05. Planned comparisons confirmed that RTs differed for the three colors in the Color group (green: 575 ms, blue: 567 ms, red: 554 ms), *F*(2,92) = 4.69, *p* = .01, *η*_p_^2^ = .09, while RT was not different for the colors in the Orientation group (green: 445 ms, blue: 444 ms, red: 444 ms), *F*(2,92) = 0.02, *p* = .98. This time this group difference was not supported by a significant Practice Group × Color interaction, *F*(2,92) = 2.08, *p* = .13.

The second ANOVA consisted of a 2 (Practice Group: Color group vs. Orientation group) × 9 (Block) × 3 (Response: pressing 1 ×, 2 ×, 3 ×) ANOVA. It was carried out to assess the effect of the number of key presses on the RT of the first key press. It showed that across all practice blocks, RT decreased with the number of ensuing key presses (520 ms, 505 ms, 490 ms, respectively), *F*(2,92) = 7.74, *p* < .001, *η*_p_^2^ = .14.

Analysis of the arcsine transformed error proportions with the above 2 × 3 × 3 × 9 ANOVA design showed by way of an Orientation main effect that error proportion was lowest for the vertical line (2.1%) and higher for the slash and backward slash (2.8% and 2.9%, respectively), *F*(2,92) = 5.47, *p* = .006, *η*_p_^2^ = .11. A marginally significant Practice Group × Orientation interaction suggested that this effect was solely caused by the Orientation group (1.9%, 3.1%, 3.2%, respectively) as it did not occur in the Color group (2.3%, 2.6%, 2.6%, respectively), *F*(2,92) = 2.59, *p* = .08, *η*_p_^2^ = .05. The Block main effect, *F*(8,368) = 3.09, *p* = .002, *η*_p_^2^ = .06, showed that error proportion decreased after Block 1 (3.3%) and was constant in Blocks 2–4 (all three: 2.3%), and then gradually increased again from Block 5 till Block 9 (from 2.6% in Block 5 to 2.9% in Block 9).

In short, RTs in the practice phase showed a larger reduction across practice blocks for the Orientation than for the Color group and that responses to vertical lines by the Orientation participants were generally faster and with marginally less errors than to the tilted lines. Responses were initiated faster as the response key was pressed more often.

#### Test phase

Reaction times of the first response from errorless trials were subjected to a mixed 2 (Practice Group) × 3 (Target Feature: Same, Other-Inconsistent, Other-Consistent) × 3 (Response: pressing 1 ×, 2 ×, 3 ×) × 2 (Block: 10 vs. 11) ANOVA with Practice Group as between-subject variable. This design circumvented the fake consistency variable in the control condition of Experiment 1 by pooling the three responses of the control condition into the ‘Same’ (as during practice) level of the Target Feature variable. The two ‘Other’ levels comprised the experimental condition and distinguished between trials in which the then irrelevant feature was consistent versus inconsistent with what participants had practiced before.

**Fig. 5:**
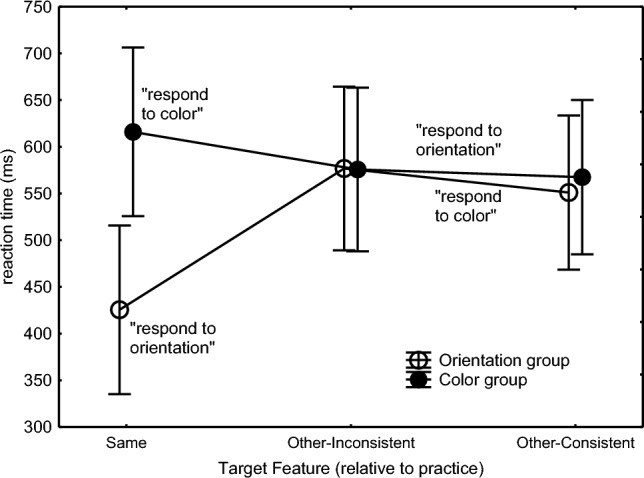
Mean reaction times in the Orientation and Color practice groups of Experiment 2 in the ‘Same’ feature (as during practice) condition and the consistent and inconsistent ‘Other’ feature conditions in test Blocks 10 and 11. Consistency refers to the irrelevant feature in the ‘Other’ test conditions. For instance, when the Color group was responding to the line orientation in the Other conditions, the required response could be consistent or inconsistent with the response given during practice to the now irrelevant line color

The Practice Group × Target Feature interaction, *F*(2,92) = 34.0, *p* < .001, *η*_p_^2^ = .43, is shown in Fig. 5 and also seemed responsible for the Target Feature main effect, *F*(2,92) = 9.82, *p* < .001, *η*_p_^2^ = .18. Planned comparisons confirmed the hypothesis that in the Orientation group RTs were longer in the Other-Inconsistent than in the Other-Consistent conditions, *F*(1,46) = 5.20, *p* = .03, *η*_p_^2^ = .10, but the hypothesis that the Color Practice group would be slowed by inconsistent colors in the Other-Inconsistent relative to the consistent colors in the Other-Consistent condition was not supported, *F*(1,46) = 0.07, *p* = .79.

Planned comparison replicated the finding in Experiment 1 that the Orientation group responded much faster to the orientation of the line in the Same condition than to its color in the Other-Consistent condition, *F*(1,46) = 19.46, *p* < .001, *η*_p_^2^ = .30. Remarkably, even the Color group was marginally faster in the new task of responding to orientation in Other-Consistent than in the familiar task of responding to color in Same, *F*(1,46) = 3.66, *p* = .06, *η*_p_^2^ = .07 (Fig. 5).

A Block × Target Feature interaction, *F*(2,92) = 12.09, *p* < .001, *η*_p_^2^ = .21, together with the Block main effect, *F*(1,46) = 36.68, *p* < .001, *η*_p_^2^ = .44, showed that the improvement from Blocks 10 to 11 was mostly due to the Other conditions (Other-Inconsistent: from 618 to 535 ms, Other-Consistent: from 598 to 521 ms) and hardly due to the Same condition (from 525 to 517 ms). The Response main effect further showed that RTs to the first key press were longer with 1 and 2 than with 3 successive key presses (1 ×: 564 ms, 2 ×: 562 ms, 3 ×: 530 ms), *F*(2,92) = 8.75, *p* < .001, *η*_p_^2^ = .16. Response did not interact with any other independent variable.

**Fig. 6:**
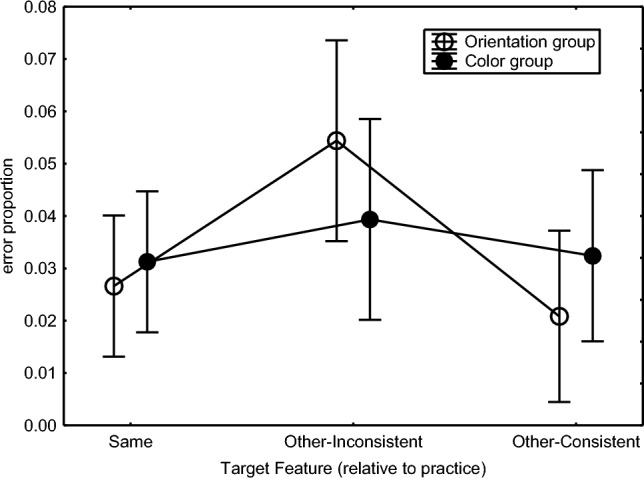
Error proportion in the Orientation and Color practice groups in the ‘Same’ feature (as during practice) condition and the ‘Other’ feature conditions of test Blocks 10 and 11 of Experiment 2 (see caption of Fig. 5)

The same ANOVA on arcsine transformed error proportions showed by way of a Group × Target Feature interaction (Fig. 6), *F*(2,92) = 4.35, *p* = .02, *η*_p_^2^ = .09, that the error proportion was especially high when the Orientation group responded to the color feature, while the line orientation was inconsistent with what they had been practicing. Planned comparison of error proportions in the Other-Consistent and Other-Inconsistent conditions confirmed this difference for the Orientation group, *F*(1,46) = 25.41, *p* < .001, *η*_p_^2^ = .36, and that it was not significant for the Color group, *F*(1,46) = 2.05, *p* = .16. A further Practice Group × Target Feature × Response interaction, *F*(4,184) = 3.55, *p* = .008, *η*_p_^2^ = .07, together with the Response main effect, *F*(2,92) = 7.56, *p* < .001, *η*_p_^2^ = .14, showed that this high error rate in the Orientation group performing in the Other-Inconsistent condition was caused primarily by pressing the key 1 time and also 2 times (Orientation group/Other-Inconsistent: 1 ×: 9.8%, 2 ×: 4.8%, 3 ×: 2.1%; Color group//Other-Inconsistent: 1 ×: 4.6%, 2 ×: 4.5%, 3 ×: 2.8%). Planned comparisons confirmed that the Consistent-Inconsistent difference was statistically significant with the 1-key press responses for the Orientation group responding to color, *F*(1,46) = 39.43, *p* < .001, *η*_p_^2^ = .46, and not for the Color group pressing the key one time when responding to orientation, *F*(1,46) = 1.88, *p* = .18.

In short, the test phase confirmed that when the Orientation group was responding to color, this response was slower when the line orientation was inconsistent than when it was consistent with earlier practice. This consistency effect was reflected also in a high error proportion when the response consisted of a single key press. No consistency effects were observed for the Color group. Responses were faster responding to line orientation than to color in the Orientation group, and even in the Color group despite their experience with responding to color features. RTs generally reduced as the response included more key presses.

#### Discussion

Despite changing the proportion of consistent trials, using responses consisting of repeatedly pressing a key, and counterbalancing responses across stimuli, Experiment 2 replicated the main findings of Experiment 1. Most importantly, the data confirmed the consistency effect for the Orientation group in that responding to the color feature was significantly slowed and involved more errors when the then irrelevant line orientation was inconsistent with the response practiced before. Again, responding to the line orientation by the Color group showed no consistency effect of the color feature.

The indication in Experiment 1 that a consistency effect was not observed in the Color group because color identification was too slow, was supported by the slower RTs in the practice and test phases for the color than for the orientation group. In fact, even after practice, the Color group responded faster to the to them unfamiliar line orientation than to the to them familiar line color in the test phase.

The consistency effect in the Orientation group that was observed in error proportion was strongest for the responses consisting of one key press. This suggests that when responding to the line color, the then irrelevant orientation feature was available rapidly enough to prime another response representation (causing the consistency effect in RT), while it also prevented inhibition of the ensuing key press during response execution (causing the consistency effect in error proportion). Instead, when the correct response included three successive key presses, the irrelevant orientation feature seems not to have inhibited responses (causing no consistency effect with 3 key press responses). Perhaps, execution of the first key press provided the time to inhibit that irrelevant feature so that it did not affect the second and/or third key press. There is indeed ample evidence from studies with discrete keying sequences that control processes may be active, while a first key press is being executed (Verwey, 1995, in press; Verwey et al., 2015). Here, this means that the effect of the then irrelevant feature could be suppressed in time with 3-press responses (Memelink & Hommel, 2013; Müller et al., 2003; Su et al., 2014), but not with 1-press responses.

Concurrent programming can explain also the finding that RT of the first key press reduced with the number of key presses while it usually increases (Canic & Franks, 1989; Sternberg et al., 1978; Verwey, 1999). This uncommon effect of the number of key presses suggests that participants programmed three successive key presses by default and adjusted this when only 1- or 2-key press responses were needed. The generally shorter RTs^1^ as well as the higher variability in Experiment 2 (Fig. 4) than in Experiment 1 (Fig. 1) suggests that the time to depress the response key for the first time, resulted from a mixture of, on the one hand, responses entirely following the decision how many times the key was to be pressed (advance response programming), and on the other hand of fast first response execution during which the number of ensuing responses is prepared and programmed (concurrent response programming).

Lastly, Experiment 2 confirmed the finding in Experiment 1 that during practice participants attended only to the target feature. This was indicated by RTs and errors of the Orientation group being different for the various orientation features (e.g., responses were faster to the vertical line only in the Orientation group) while they were not for the Color group. Vice versa, RTs of responses to color in the Color group were significantly different while they were not in the Orientation group. So, these results confirm that during practice participants of both groups ignored the irrelevant stimulus feature. The consistency effect of the Orientation group indicates that, at least in that group, practice makes processing of the practiced feature mandatory.

## Experiment 3

Experiments 1 and 2 showed evidence for the development of associations between the orientation feature and the response in the Orientation group. These line orientation–response associations had been induced by consistently responding to the line orientation during practice while varying the line color prevented associations between a color and the response. This raised the question whether the simultaneous manipulation of line and color prompts independent associations at the same time between both stimulus features and the response, or whether in that situation associations develop between integrated stimulus and response representations because the various stimulus features are first integrated into a unified stimulus representation (i.e., the classic S–R model).

Experiment 3 tested this by having participants practice responding to three so-called *compound stimuli* that always consisted of the same line color and line orientation combinations, namely a blue ‘|’, a green ‘\’, and a red ‘/’. Like in Experiment 2, responses consisted of pressing a key 1, 2 or 3 times. The test phase included an Orientation condition and a Color condition, each including all 9 combinations of three orientation and three color features. In the Orientation test condition, participants responded to the line orientation, and were confronted with colors consistent and inconsistent with the color observed with that line orientation during prior practice. In the Color test condition, the same participants responded to the stimulus color while line orientation could be consistent or inconsistent with the orientation practiced before. The question was whether after practicing with fixed color–orientation combinations consistency of the irrelevant color and of the line orientation features would still affect RTs.

The *compound stimulus representation hypothesis* postulates that practicing with fixed color–orientation combinations induces an integrated stimulus representation incorporating those features. Then, orientation and color would both have to be identified to activate the compound stimulus representation and with practice this representation would become associated with the response given during practice. This hypothesis essentially constitutes the classic notion that, after practice in a particular task, associations develop between the full stimulus representation and the full response representation (Logan, 1988; Teichner & Krebs, 1974; Welford, 1968). For Experiment 3, this would imply that response skill does not transfer to a stimulus with a novel combination of familiar features, and that performance would benefit from practice only when both the color and orientation features of the line stimuli are consistent with practice.

Instead, the *independent feature hypothesis* postulates separate feature–response associations and, hence, that the response is selected on the basis of an association between the fastest available stimulus feature and the response (or the still unspecified response feature). The more slowly available stimulus feature may still slow the first response if available only slightly later, or it may have no effect when the response has already been selected. This hypothesis is in line with the DA model which assumes that each feature-specific module has its own response selection mechanism and that attention determines which module, and hence stimulus feature–response association, is being used (Magen, 2019; Magen & Cohen, 2002). The independent feature hypothesis is consistent also with visual search studies that indicated that participants can pay attention to a specific stimulus feature and suppress the influence of irrelevant features (Memelink & Hommel, 2013; Müller et al., 2003; Su et al., 2014). According to the independent feature hypothesis, the indications in Experiments 1 and 2 that the color feature is available later than the orientation feature predict for the Color test condition a clear effect of line orientation consistency. Instead, when participants respond to line orientation in the Orientation test condition consistency with the practiced color may not affect response speed because it is available only after the response has already been selected. It may, however, still affect error proportion because responses with several key presses can be adjusted while the first key press is being executed.

### Method

**Fig. 7:**
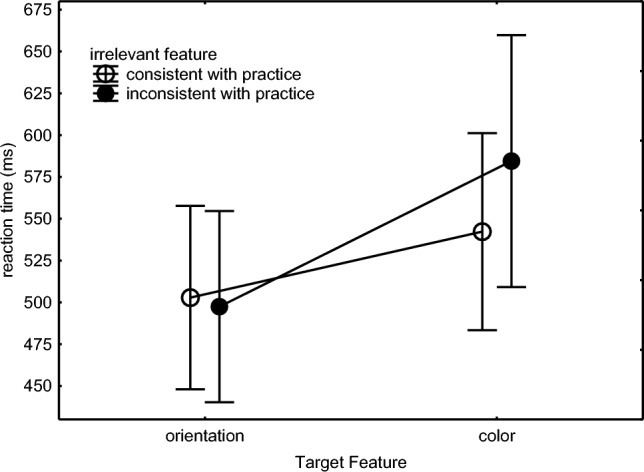
Mean reaction times across Blocks 10 and 11 of the test phase of Experiment 3 for participants responding to the line orientation or the line color while the other, irrelevant feature (color or orientation, respectively) was either consistent or inconsistent with practice

Experiment 3 was identical to Experiment 2, using the same PCs and keyboards, except that there was only one practice group, and this group responded with repeated pressing of the G key to a blue ‘|’, a green ‘\’, and a red ‘/’. The three responses were counterbalanced across participants so that overall a specific response was not associated with a particular stimulus. The 24 participants consisted of 20 females and 4 males (18–25 years old, mean 20.8). A post hoc power analysis using the effect size of the important Target Feature × Consistency interaction in Fig. 7 showed that with 5 participants per group (given .05 alpha, and two-tailed testing) a power would have been reached of 0.95 for detecting this effect. Each block in the test phase included an Orientation condition subblock and a Color condition subblock. Subblock order was counterbalanced across the participants within each test block, and each of the subblocks contained 18 consistent and 36 inconsistent trials. Every participant worked for approximately 120 min.

### Results

#### Practice phase

The RTs of the first key press in the errorless trials in Blocks 1 through 9 were analyzed with a 3 (Response: pressing 1 ×, 2 ×, 3 ×) × 9 (Block) ANOVA. It showed the expected main Block effect, *F*(8,184) = 11.67, *p* < .001, *η*_p_^2^ = .34. A main Response effect confirmed the finding in Experiment 2 that the first response was faster with more ensuing key presses (463 ms, 436 ms, 427 ms, respectively), *F*(2,46) = 33.01, *p* < .001, *η*_p_^2^ = .59. A 3 (Stimulus: blue ‘|’, green ‘\’, red ‘/’) × 9 (Block) ANOVA showed that responses were fastest for the blue ‘|’ (431 ms), slower for the red ‘/’ (445 ms), and slowest for the green ‘\’ (450 ms), *F*(2,46) = 4.10, *p* = .02, *η*_p_^2^ = .15.

The 3 (Response) × 9 (Block) ANOVA on arcsine transformed error proportions showed by way of a significant Block × Response interaction, *F*(16,368) = 2.06, *p* = .009, *η*_p_^2^ = .08, that error proportion gradually reduced for the responses consisting of 2 key presses while it gradually increased for those with 1 and 3 key presses (Block 1: 2.0%, 3.9%, 2.5%; Block 9: 3.4%, 2.0%, 3.3%). The 3 (Stimulus: blue ‘|’, green ‘\’, red ‘/’) × 9 (Block) ANOVA assessed potential errors differences between the three stimuli but these were not significant.

So, the practice phase replicated that RT reduced as the response involved more key presses, and showed that RTs were shortest for the blue ‘|’ and longest for the green ‘\’.

#### Test phase

RTs of the first key press were analyzed with a 2 (Target Feature: Orientation condition vs. Color condition) × 2 (Consistency: of the irrelevant feature) × 3 (Response: pressing 1 ×, 2 ×, 3 ×) × 2 (Block: 10 vs. 11) ANOVA. The significant Target Feature × Consistency interaction (Fig. 7), *F*(1,23) = 16.21, *p* < .001, *η*_p_^2^ = .41, which also seem to have caused the main effects for Target Feature, *F*(1,23) = 15.06, *p* < .001, *η*_p_^2^ = .40, and Consistency, *F*(1,23) = 5.55, *p* = .03, *η*_p_^2^ = .19, showed that consistency of the irrelevant stimulus feature with prior practice affected RT when responding to color and not when responding to orientation. Planned comparisons showed that when the target feature was consistent with practice, RT was longer when responding to color than to orientation, *F*(1,23) = 6.41, *p* = .02, *η*_p_^2^ = .22. In addition, the Response main effect showed that RTs of the first response reduced as it was followed by more key presses (550 ms, 530 ms, 515 ms, respectively), *F*(2,46) = 5.55, *p* = .007, *η*_p_^2^ = .19. Response did not interact with any other variables. The Block main effect showed improvement across the two successive test blocks (553 ms vs. 510 ms), *F*(1,23) = 20.15, *p* < .001, *η*_p_^2^ = .47.

The same design as mentioned above with the RTs was used to analyze arcsine error proportions. It showed that more errors were made in the inconsistent than the consistent condition (5.1% vs. 2.3%), *F*(1,23) = 27.35, *p* < .001, *η*_p_^2^ = .54. A marginally significant Target Feature × Consistency interaction revealed that this consistency effect tended to be larger in the Color than in the Orientation condition, *F*(1,23) = 3.30, *p* = .08, *η*_p_^2^ = .13. Still, planned comparisons showed that the effect of consistency due to the irrelevant line orientation feature was not only significant in the Color condition (consistent orientation: 2.2% vs. inconsistent orientation: 6.1%), *F*(1,23) = 17.21, *p* < .001, *η*_p_^2^ = .43, but importantly the irrelevant color feature also affected error proportion in the Orientation condition (consistent color: 2.3% vs. inconsistent color: 4.1%), *F*(1,23) = 10.57, *p* = .004, *η*_p_^2^ = .31.

Hence, the test phase showed that responding to the color feature was slowed and involved more errors when the line orientation was inconsistent with practice. When responding to the line orientation, inconsistency of the irrelevant color did not slow responses but importantly error proportion was significantly higher when the color was inconsistent with practice. Experiment 3 further replicated the slower responses to the color than to line orientation features and the shorter RTs as responses included more key presses.

### Discussion

The test phase of Experiment 3 showed a consistency effect in RTs and errors in the Orientation condition and this time also in the errors observed in the Color condition. These results support the independent feature hypothesis and demonstrate for the first time that in addition to line orientation-response associations, suggested already in Experiments 1 and 2, color-response associations developed too. That the consistency effect was observed in the Orientation condition only in terms of error proportion is in line with a slower identification of the color than of the orientation feature. The results reject the classic compound stimulus representation hypothesis that assumes that stimulus features are first unified and only then as compound stimulus trigger the response. That this was observed after practice with compound stimuli suggests that practicing with these stimuli still prompts independent feature–response associations. Hence, even though during practice RT was probably based on the rapidly available orientation feature the perceived color feature became associated with the response as well.

## General discussion

The present study was aimed at testing the stimulus feature–response association hypothesis which posits that with practice associations develop at the lowest levels possible of the perceptual and motor hierarchies. In the present experiments, this concerned associations between the orientation or the color features of a line stimulus and the responses practiced with. All three experiments confirmed the predicted development of a line orientation–response association. This was shown by slowed and less accurate responding to stimulus color when the then irrelevant line orientation was inconsistent with practice and primed another than the required response. Support for the development of a color–response association was shown in Experiment 3 by a higher error proportion when responding to the orientation of the line stimulus, while its color was inconsistent with the required response.

The limited support for color–response associations can be attributed to a slower identification of color than of line orientation features. This explanation is supported by the repeated finding in all three experiments that responding to color was generally slower than to line orientations. This refutes the assumption in the Introduction that color and orientation would take similar processing times because they are of a comparable simplicity (Su et al., 2014). Slower color identification can also explain that when responding to line orientation in Experiment 2 inconsistent colors showed the consistency effect primarily with single-key press responses. Namely, the color identity seems to have become available shortly after the orientation identity so that it could still interfere with the execution of the selected single key press while this conflict was inhibited in the case of 3 key presses. Evidence for control processes concurring with execution of a key press has been shown many times with discrete keying sequences (Verwey, 1995, in press; Verwey et al., 2015). In any case, the higher error proportion observed with inconsistent trials in the Orientation condition of Experiment 3 indicates that color–response associations develop too.

In retrospect, the indications that stimulus features can be used for selecting responses seems related to additive factors studies showing that manipulation of the number of alternatives not only influenced the response selection processing stage but also the extraction of stimulus feature stage while manipulating S–R compatibility influenced the stimulus identification stage too (Sanders, 1990). Furthermore, the indication for the independent use of color and orientation features is in line also with the intentional weighting of features like color and orientation feature dimensions (Memelink & Hommel, 2013; Müller et al., 2003; Su et al., 2014) and that this involves the attentional focusing suggested by the DA model (Magen, 2019; Magen & Cohen, 2002). As mentioned, the DA model assumes that the visual system contains separate modules for the various stimulus features and that response selection occurs within the active task module. Experiments 1 and 2 support the assumption of the DA model that a conflict between different modules is prevented by attention determining in advance the activity of these modules. That is, these two experiments showed that in the practice phase the Orientation group had different RTs and errors for the three line orientations and not for the three colors. Conversely, the Color group in the practice phase of both experiments showed different error proportions for the three colors, and for RTs in Experiment 2, but not for the various line orientations during practice. In addition, the present results also suggest that even after practice, these modules can be independently available. This is consistent with visual search studies showing that after 40–50 min of practicing unique combinations of orientation and color features (e.g., a green horizontal bar among green vertical and red horizontal bars) transferred to a new target also if that involved just the same color or just the same orientation (Su et al., 2014).

The repeated finding that participants could ignore the irrelevant features during practice but not after practice when responding to the other feature confirms the distinction between permanent associations that are task-specific but not under deliberate control, and temporary bindings that can be deliberately controlled (Herwig & Waszak, 2012; Hommel, 2019; Hommel & Colzato, 2009). The fact that the consistency effect occurred at all shows that the associations between stimulus features and responses that develop during practice became permanent in the context of the present task and were beyond voluntary control. Instead, the indications that during practice the irrelevant features were ignored indicates that with little practice and depending on the task, they may be temporarily bound to the responses.

These findings corroborate that what once was assumed to consist of a single association between a stimulus and a response representation (Logan, 1988; Teichner & Krebs, 1974; Welford, 1968), can now be argued to consist of a task-specific mixture of associations that includes individual stimulus features that have been attended to during practice and response representations. According to the claim of the stimulus feature–response association hypothesis that this involves the lowest possible levels of the perceptual and also of the motor hierarchy, future research can test the prediction that on the response side this would involve especially the feature (i.e., the response parameter) that distinguishes the alternative responses. The other response features (i.e., the generalized motor program and fixed parameters) can be assumed to have already been prepared (Ansorge et al., 1998; Neumann, 1990).

The notion that with practice associations develop between stimulus features and features of responses seems incongruent with the proposal by TEC that responses are represented by, and selected via, codes of their perceptual effects. However, the binding construct of TEC is largely based on studies with limited practice (Hommel, 2019). It is quite possible that with substantial practice task-specific associations (shortcuts) develop that directly trigger the required response after detecting a specific stimulus feature rather than that response representations are assessed via their expected action effects.

In conclusion, the present experiments support the development of associations with practice between individual stimulus features and responses. This was clear in Experiments 1 and 2 for orientation feature–response associations but the identification of color features seems to have been too slow to outrun responding on the basis of temporary line orientation-response bindings. However, Experiment 3 indicated that color feature–response associations develop too and that these associations may in the case of the serial key pressing responses still affect later responses. The results suggest that participants can initially choose to prepare stimulus feature–response bindings but that after repeatedly attending to a specific stimulus feature during practice task-specific feature–response associations develop. This suggests that the RT reduction that characterizes response selection skill can be attributed in part to the declining need with practice to wait for identification of slower stimulus features. Also, associations between stimulus and response features may operate faster than the temporary bindings that include action effects. Future research should provide more support for the stimulus feature–response association hypothesis by showing additional evidence for color–response associations when they are not overshadowed by the faster stimulus orientation features. That research should further show that stimulus features may also become associated with low level motor parameters, like absolute movement time and absolute force (cf. Neumann, 1990; Schmidt, 1975; Shea & Wulf, 2005), hence bypassing the expected sensory effects of an action.

## Footnotes

1 A 2 (Experiment 1 vs. 2) × 2 (Practice Group) × 3 (Color) × 3 (Orientation) ANOVA with Experiment and Practice Group as between-subject variables compared RTs in the last, ninth, practice block. The Experiment main effect showed that RTs were shorter when repeatedly pressing a single key in Experiment 2 than when pressing one of three alternative keys in Experiment 1 (481 vs. 560 ms), F(1,92) = 5.29, p = .02, ηp2 = .05. This ANOVA also showed that the 115 ms longer time taken to identify colors than line orientations in the last practice block (578 ms vs. 463 ms), F(1,92) = 11.08, p = .001, η_p_^2^ = .11, was according to a non-significant Experiment x Practice group interaction similar in the two experiments, F(1,92) = 0.57, p = .45.
